# Capsulectomy at Explant: Rethinking Spinal Cord Stimulator Infected Generator Pocket Management in the Immunocompromised—A Case Series

**DOI:** 10.1111/papr.70212

**Published:** 2026-10-06

**Authors:** Tigran Kesayan, Vida Motamedi, Jessica Hanflink, Patrick Assi, Salam Kassis

**Affiliations:** ^1^ Department of Anesthesiology Vanderbilt University Medical Center Nashville Tennessee USA; ^2^ Department of Neurology Vanderbilt University Medical Center Nashville Tennessee USA; ^3^ Department of Plastic Surgery Vanderbilt University Medical Center Nashville Tennessee USA; ^4^ Nova Southeastern University College of Osteopathic Medicine Fort Lauderdale Florida USA

## Abstract

**Background:**

Surgical site infections (SSI) associated with spinal cord stimulation (SCS) implants pose risks of morbidity, mortality, and increased healthcare costs. Immunocompromised patients are especially vulnerable due to impaired immune responses, delaying diagnosis and increasing complications. Current guidelines recommend SCS explantation for deep or inadequately treated superficial SSI, though the role of capsulectomy remains underexplored despite its use in other fields like plastic surgery and cardiology.

**Case Presentation:**

We present two cases of immunocompromised patients with SSI treated with SCS explantation and capsulectomy. Case 1 involved a 64‐year‐old woman with ulcerative colitis and psoriatic arthritis treated with immunosuppressive agents who developed fever and myalgia and had normal imaging. Despite treatment with a course of antibiotics, she developed granulation tissue at the midline incision which tracked to the lead anchors. Explantation revealed necrotic tissue, and capsulectomy of the implanted pulse generator pocket and around lead anchors was performed. *Staphylococcus schleiferi* , likely linked to pet exposure, was identified. Case 2 involved a 52‐year‐old woman with postlaminectomy syndrome and rheumatoid arthritis on immunosuppressants, who presented with erythema and wound dehiscence weeks post‐implant. Explantation and capsulectomy were performed to remove fibrotic tissue potentially harboring biofilm. MRSA infection was identified. Targeted antibiotics facilitated recovery in both cases.

**Conclusion:**

These cases suggest capsulectomy during SCS explantation may mitigate complications, particularly in immunocompromised patients. Current guidelines recommend capsule debridement but do not routinely address capsulectomy; its use may improve infection resolution in high‐risk populations, perhaps by eliminating biofilm associated with the capsule.

## 1 Introduction

Spinal cord stimulation (SCS) is a well‐established treatment for several painful conditions.

Surgical site infections (SSI) associated with SCS implants pose a challenge to health care professionals, increase risk of patient morbidity and mortality, and account for significant healthcare expenditure [1, 2, 3, 4, 5, 6]. SCS infection rates range from 2.4% to 3.1% [1, 5, 7]. Delay in identification and treatment of infections associated with SCS may lead to meningitis, abscess formation, and osteomyelitis [7]. Hospitalization for an SCS related infection is associated with a 1.83% mortality rate [7].

SSI risk is higher in those who are immunocompromised or are being treated with medications which impair wound healing [8]. Immunocompromised patients have an impaired ability to mount a normal local or system immune response to inform the health care professional of SSI [8]. The Neurostimulation Appropriateness Consensus Committee (NACC) Recommendations for Infection Prevention and Management as well as the Device Evaluation, Treatment, and Explantation Recommendations (DETER) review of best practices of managing neuromodulation device infections recommend explantation of an infected implant if the SSI is deep, or a superficial SSI is not adequately treated with antibiotics [3, 6].

In fields of medicine where implants are common, such as plastic surgery and cardiology, capsulotomy with explantation of an infected implant has been discussed [9, 10]. The 2025 American Society of Regional Anesthesia (ASRA) Pain Medicine consensus guidelines on infection control marked the first time that capsule debridement during SCS explantation was recommended in the chronic pain literature. Although capsulectomy was mentioned in these ASRA guidelines, no published reports currently describe this practice in SCS.

We present two immunocompromised patients with concern for SCS SSI who were treated with SCS system explantation with a capsulectomy, and propose consideration of this technique in high‐risk populations.

## 2 Case Reports

This case series does not include identifiable patient information and is therefore exempt from IRB review under Vanderbilt University Medical Center IRB policy. Consent was obtained from both patients for the publication of these cases.

### 2.1 Patient 1

A 64‐year‐old woman with ulcerative colitis (UC) and psoriatic arthritis (PsA) is being treated with upadacitinib (Rinvoq) and mycophenolate, who underwent SCS implantation for chronic lumbar radiculopathy. She was given 2 mg IV cefazolin within 60 min prior to incision. The Disease‐modifying antirheumatic drugs (DMARD), upadacitinib, were held for 7 days prior to implant, and mycophenolate was held for one week prior to implant with instructions to resume one week postoperatively. Three weeks postoperatively she developed a fever, myalgias, leukocytosis, and pain over the IPG site. She had a CT scan of the thoracolumbar spine, and there were no concerning findings for infection on this imaging study. She was started on a 14 day antibiotic therapy with dual coverage for methicillin resistant Staph aureus (MRSA), and her symptoms quickly improved. A consult request was placed for Infectious Disease (ID); however, she preferred not to be evaluated by ID. Thirteen weeks postoperatively she noticed a bump at the midline incision site. Examination revealed granulation tissue (Figure 1A).

**FIGURE 1:**
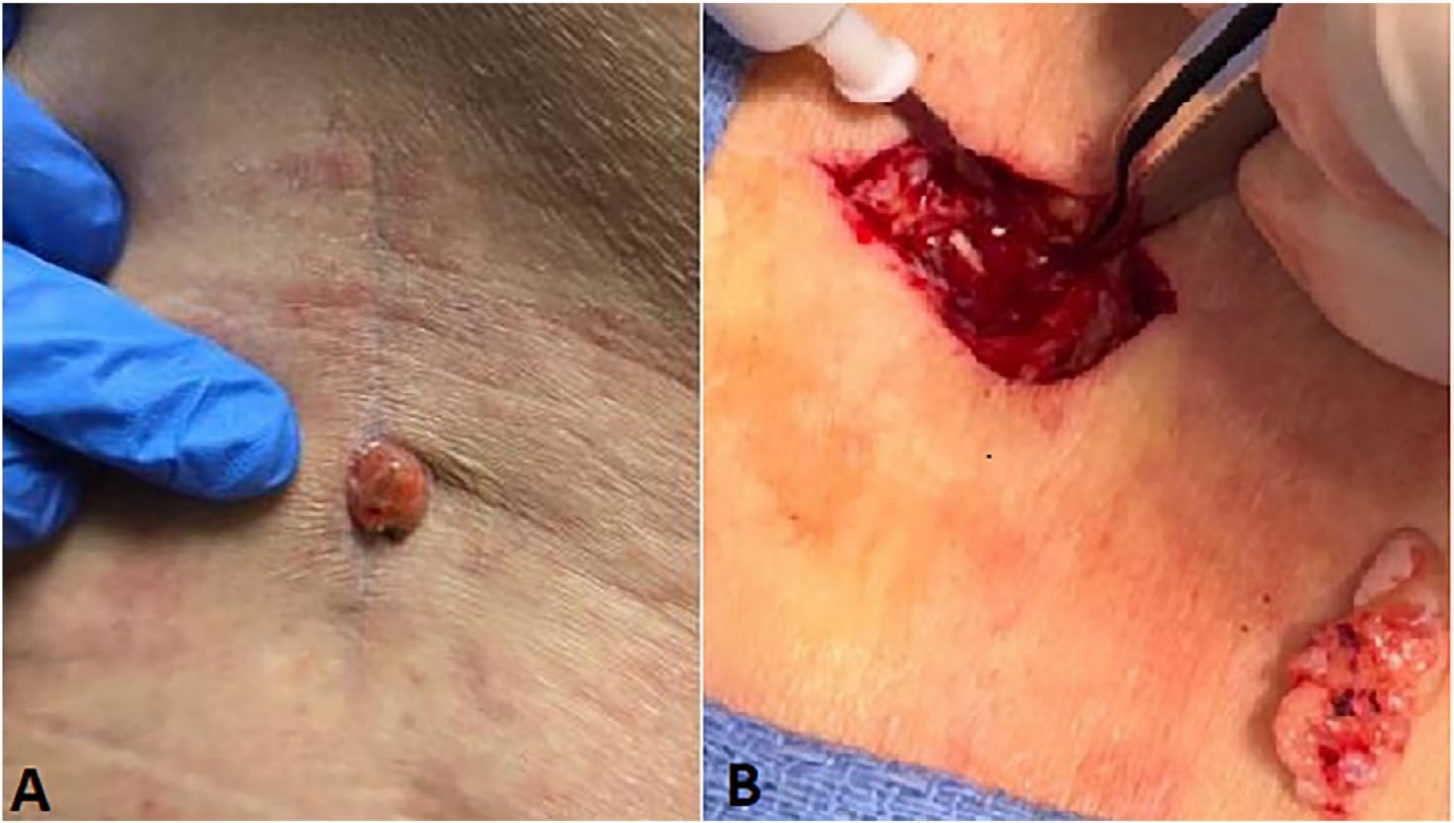
(A) Granulation tissue noteed on examination. (B) Grandulation tissue resected, note the white plastic of the SCS lead anchor.

There was no surrounding redness or discharge or warmth to the touch. She denied any fevers or myalgia. She was evaluated by a plastic surgeon, and examination revealed granulation tissue which traced down to the SCS lead anchors (Figure 1B). The anchors were swabbed for pathology. There was concern for infection with a weakened immune response given the patient's immunocompromised state. She was started on empiric antibiotics and scheduled for explantation 3 days later.

She underwent explantation of the SCS system with capsulectomy of the implanted pulse generator (IPG) pocket, capsulectomy around the anchors, and excisional debridement of skin and subcutaneous tissues with a complex closure of the wound a by the pain physician in conjunction with a plastic surgeon. During surgery there was a larger area of deeper necrotic tissue than was suspected based on her physical examination findings preoperatively. These intraoperative findings informed our multidisciplinary team's decision to perform a capsulectomy and debridement. Fluid from the infected pocket site grew *Staphylococcus schleiferi* , a bacteria usually found in household pets [11]. The patient was caring for an ill cat with a skin infection at the time of her implant. She improved with five days of postoperative antibiotics and has been well since. Unfortunately, her radicular pain has returned since her SCS explantation.

### 2.2 Patient 2

Patient 2 is a 52‐year‐old woman with a history of postlaminectomy syndrome following lumbar decompression and fusion, left lower extremity amputation as a consequence of a prior postoperative MRSA infection, and rheumatoid arthritis treated with hydroxychloroquine and golimumab (Simponi) presented for evaluation of SCS for the treatment of postlaminectomy syndrome. The patient was receiving the intravenous formulation of DMARD golimumab (Simponi Aria), which is administered every 8 weeks by a healthcare provider. She received her last dose of golimumab approximately 9 weeks prior to surgery, consistent with holding the medication for one full dosing interval.

After a positive SCS trial she underwent SCS implantation. She was given IV Vancomycin 15 mg/kg prior to incision. At the six week postoperative visit she reported 80% improvement in pain intensity. Seven weeks postoperatively she noticed some slight swelling over the IPG pocket site. On examination there was slight erythema around the incision site. Given her immunocompromised state and lower threshold for SSI, she was started on empiric treatment for MRSA. The following week, 8 weeks postoperatively, the erythema was resolved; however, there was a small area where the midline incision was starting to dehisce (Figure 2). Given her immunocompromised state and prior complications from a postoperative MRSA infection which did not respond to extensive antibiotic therapy and led to a below the knee amputation, the decision was made to explant the SCS system. The SCS IPG, anchors, and leads were explanted along with a capsulotomy of the fibrous capsule around the IPG within the IPG pocket as well the capsule around the lead anchors by the pain physician (Figure 3). The wound bed was extensively irrigated with antibiotic impregnated normal saline, and the same layer of the pocket was extended by about 2 cm circumferentially for tension free wound closure by the pain physician. Fluid from the pocket site grew MRSA; she was successfully treated with 7 days of postoperative antibiotics.

**FIGURE 2:**
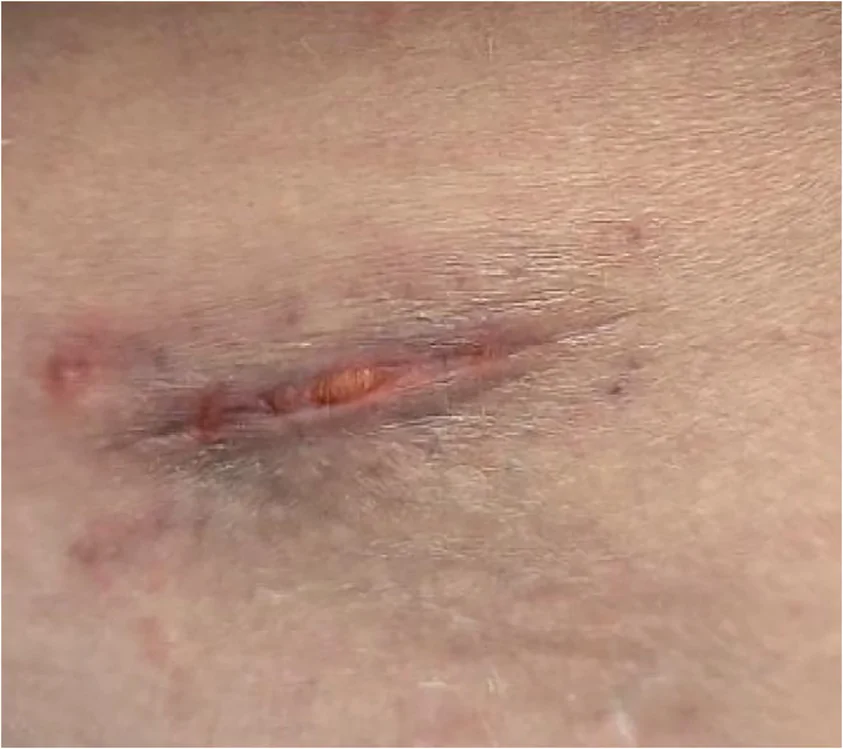
IPG site for Patient 2 during pre‐operative visit for consideration of SCS explanation demonstrating an area of wound dehiscence.

**FIGURE 3:**
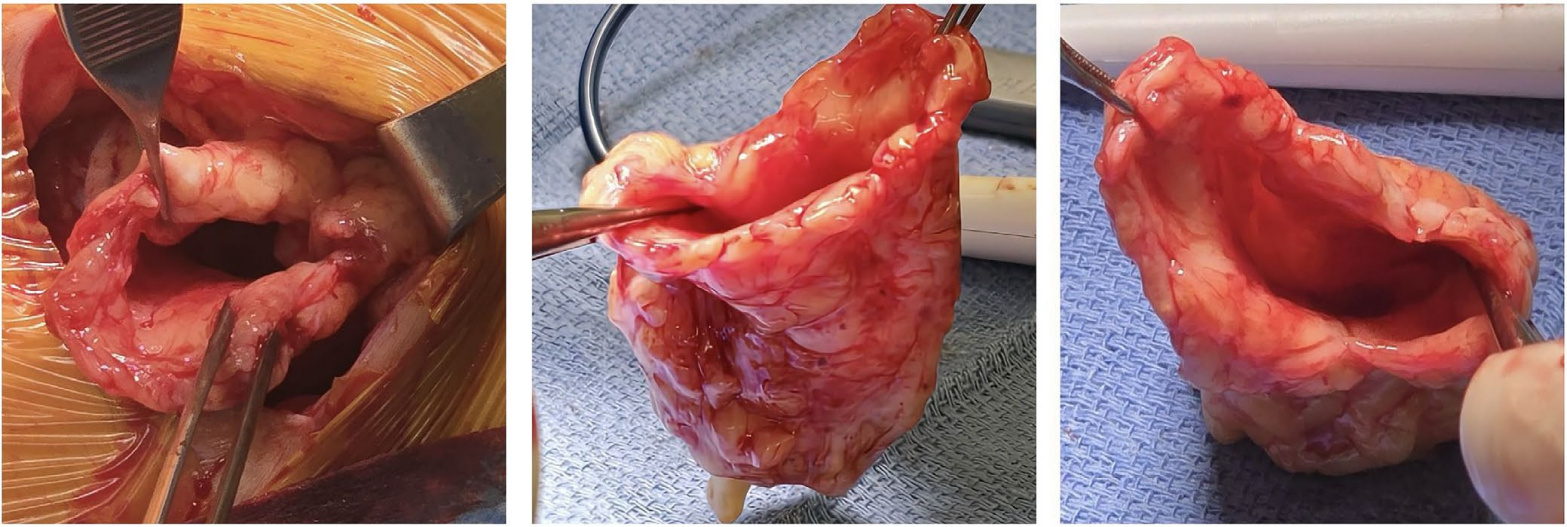
Explanted fibrosed IPG pocket capsule from Patient 2.

### 2.3 Technique for Capsulectomy

Capsulectomy generally involves circumferential incision and removal of the fibrous capsule surrounding the implanted pulse generator and lead anchors. The capsule is typically dissected free using sharp and blunt techniques until the fibrotic tissue is completely removed, as was described in previous studies for breast implant illness [9]. The goal is to remove the capsule in its entirety to eliminate tissue that may harbor biofilm. The wound bed is then irrigated with antibiotic‐impregnated normal saline prior to closure. Similar techniques have been described in breast implant and cardiac literature, the latter defining capsule debridement as removing more than two‐thirds of the fibrous capsule around a device and leads [9, 10].

## 3 Discussion

Two patients with SSI of the SCS systems were treated with explantation of the SCS system and capsulectomy of the fibrous capsule around the SCS IPG and the anchors. The 2025 ASRA guidelines for regional anesthesia and pain medicine recommend that in the case of SCS infection which is to be treated with explantation, to remove the infected hardware and debride all necrotic and fibrotic tissue including the capsule, followed by extensive irrigation [1]. Although there are no prior reports which support the excision of the capsule around the implant, rather than debriding the capsule, we argue that a capsulotomy would lead to a higher chance of removing biofilm and the risk of future infection complications, especially in those who are immunocompromised.

Fibrous capsule formation with collagenous tissue around an implant is triggered by a foreign body reaction and starts almost immediately after implantation of a foreign body [12]. Capsules can range from micrometers to 10 mm in thickness [12]. Free‐living, or planktonic, bacteria come into contact with solid surfaces of foreign implanted material and initiate the formation of biofilm [12, 13]. Biofilm leads to increased antibiotic resistance by multiple mechanisms [14]. Biofilm also impairs host immune response to the local infection, making it an even bigger concern for an immunocompromised host [13]. Debriding the capsule at SCS explantation without a complete capsulotomy increases the risk of leaving behind remnants of biofilm.

Capsulectomy with breast implant explantation is recommended in those with capsular contracture which is thought to be caused by biofilm formation, as well as for Breast Implant Illness (BII), a set of systemic symptoms associated with breast implants [9, 12]. In a retrospective cohort study of 200 patients with BII, 96% had improvement or resolution of their symptoms after explantation and capsulotomy. 69% of the patients had a positive microbial culture, and all of them improved after antibiotic treatment [9].

In those with cardiac implantable electronic device (CIED), the role of capsulectomy at time of explantation or exchange is not clear. A retrospective review of 773 patients who underwent CIED replacements, those who had a capsulotomy of the capsule from the old implant had a lower risk of infection with the new implant [10]. However, a prospective randomized trial of 195 patients undergoing CIED generator replacement did not show a decreased rate of future infection in those who had a capsulotomy compared to those who did not [15]. It is not clear if any of the participants were immunocompromised.

Both of our patients were being treated with immunosuppressive agents for their rheumatologic disease, and these agents may have played a role in impaired wound healing and creating a higher risk for infection. For Patient 1, upadacitinib was held for 7 days prior to implant, and mycophenolate was held for 7 days. For Patient 2, golimumab was held for > 8 weeks prior to the initial implant. These timelines were informed by guidelines set forth by ASRA and the American College of Rheumatology/American Association of Hip and Knee Surgeons [1].

Management of SCS SSI may depend on depth of infection. Superficial SSI involve the skin and subcutaneous tissues, while deep SSI involve the deep soft tissues like the muscle and fascia [3, 6]. Superficial infection at the epidermis and dermis layers frequently presents with localized tenderness and erythema, often do not have purulent exudate and are not associated with an underlying abscess, and usually respond well to oral antibiotics [6]. With prompt and appropriate antibiotic treatment, the SCS implant can be salvaged in those with superficial SSI [3, 6]. In cases of deep SSI, SCS explantation is often recommended along with appropriate antibiotic treatment [1]. The immunocompromised patient his at risk of an impaired ability to mount an immune response to raise clinical suspicion of a deep SSI or an extensive superficial SSI with tracking from the IPG pocket site toward midline along the leads. There should be a lower threshold to treat a superficial SSI with SCS explanation in the immunocompromised host.

The health care provider should consider closer follow up and a lower threshold for SCS explantation in an immunocompromised patient who may have only minimal symptoms of a superficial SSI. Imaging studies may also not show findings associated with SSI in those who are immunocompromised, such as in our Patient 1. Interestingly, imaging may be normal in those with SCS SSI up to a third of the time in those who are not necessarily immunocompromised [16].

It is important to mention that it is possible these patients would have improved with explanation without necessarily performing a capsulotomy. WE had concerns that just the explant and post‐operative antibiotics may possibly not be enough. The decision to perform a capsulectomy was based on the patient's immunocompromised state, findings during the explanation surgery, and the concern for biofilm and concerns with clearing indolent infections discussed above.

Overall, these two cases highlight the potential role of capsulectomy during SCS explantation in those with SCS SSI, especially in immunocompromised patients who have impaired clearing of indolent infections. Although current SCS explantation guidelines do not routinely recommend capsulectomy, it should be considered, particularly in immunocompromised patients. Further studies are warranted to evaluate its effectiveness in infection resolution and long‐term outcomes. Our case series also highlights the importance of a multidisciplinary approach for the treatment of SCS if explantation is required, such as collaboration with colleagues from other surgical specialties.

## Author Contributions

All listed authors have contributed to the manuscript substantially, in the planning of the manuscript, the writing of the manuscript, as well as the editing of the manuscript. Dr. Kesayan and Dr. Kassis performed the procedures listed in the manuscript.

## Funding

The authors have nothing to report.

## Ethics Statement

The authors have nothing to report.

## Consent

Consent was obtained.

## Conflicts of Interest

The authors declare no conflicts of interest.

## Data Availability

The data supporting the findings of this report are subject to privacy restrictions. De‐identified participant data are available from the corresponding author upon reasonable request.
